# Measuring anhedonia: impaired ability to pursue, experience, and learn about reward

**DOI:** 10.3389/fpsyg.2015.01409

**Published:** 2015-09-17

**Authors:** Kristine Rømer Thomsen

**Affiliations:** Centre for Alcohol and Drug Research, Department of Psychology and Behavioural Sciences, Aarhus University, Aarhus C, Denmark

**Keywords:** anhedonia, reward, pleasure, motivation, learning, depression, schizophrenia, addiction

## Abstract

Ribot’s (1896) long standing definition of anhedonia as “the inability to experience pleasure” has been challenged recently following progress in affective neuroscience. In particular, accumulating evidence suggests that reward consists of multiple subcomponents of wanting, liking and learning, as initially outlined by Berridge and Robinson (2003), and these processes have been proposed to relate to appetitive, consummatory and satiety phases of a pleasure cycle. Building on this work, we recently proposed to reconceptualize anhedonia as “impairments in the ability to pursue, experience, and/or learn about pleasure, which is often, but not always accessible to conscious awareness.” (Rømer Thomsen et al., 2015). This framework is in line with Treadway and Zald’s (2011) proposal to differentiate between motivational and consummatory types of anhedonia, and stresses the need to combine traditional self-report measures with behavioral measures or procedures. In time, this approach may lead to improved clinical assessment and treatment. In line with our reconceptualization, increasing evidence suggests that reward processing deficits are not restricted to impaired hedonic impact in major psychiatric disorders. Successful translations of animal models have led to strong evidence of impairments in the ability to pursue and learn about reward in psychiatric disorders such as major depressive disorder, schizophrenia, and addiction. It is of high importance that we continue to systematically target impairments in all phases of reward processing across disorders using behavioral testing in combination with neuroimaging techniques. This in turn has implications for diagnosis and treatment, and is essential for the purposes of identifying the underlying neurobiological mechanisms. Here I review recent progress in the development and application of behavioral procedures that measure subcomponents of anhedonia across relevant patient groups, and discuss methodological caveats as well as implications for assessment and treatment.

## Introduction

The generally accepted understanding of the term anhedonia has remained almost unaltered since Ribot (1896) first defined it as the “inability to experience pleasure” over a century ago. However, during the last 5 years the term has been subject to debate and some progress has been made in terms of elucidating the underlying neurobiological mechanisms. A number of recent reviews (Treadway and Zald, 2011; Der-Avakian and Markou, 2012; Whitton et al., 2015), summarize this progress and offer improved understanding of the underlying neurobiology. However, their conceptual understanding of anhedonia diverge. Treadway and Zald (2011) made a convincing case to differentiate between motivational and consummatory types of anhedonia and introduced the term decisional anhedonia to emphasize the influence of anhedonic symptoms on decision-making. In contrast, Der-Avakian and Markou (2012) recently argued that deficits in motivational and decision-making processes (albeit disturbed, e.g., in depressed patients) should not be labeled under the umbrella of anhedonia.

Overall, findings from affective neuroscience have challenged Ribot’s (1896) definition, which is restricted to subjectively experienced pleasure. Accumulating evidence suggests that reward consists of multiple subcomponents and processes of wanting, liking and learning (Robinson and Berridge, 2003; Berridge and Kringelbach, 2008) and these processes have been proposed to relate to appetitive, consummatory and satiety phases of a pleasure cycle (Kringelbach et al., 2012). Building on this work, we recently proposed to reconceptualize anhedonia as “impaired ability to pursue, experience and/or learn about pleasure, which is often, but not always accessible to conscious awareness” (Rømer Thomsen et al., 2015, p. 2).

**FIGURE 1:**
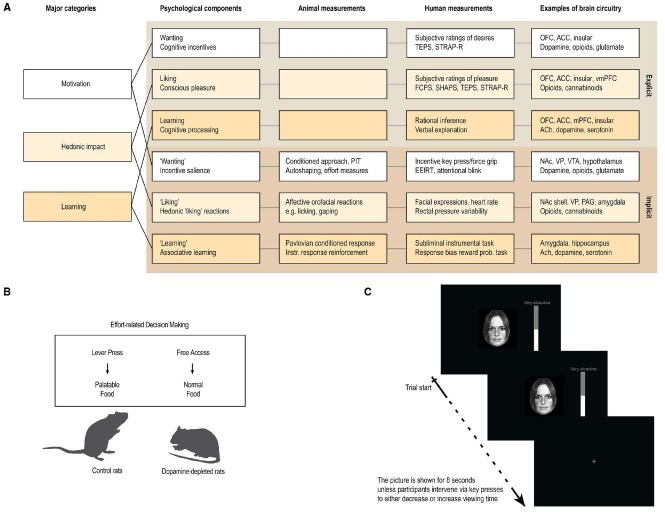
**Measuring anhedonia. (A)** Anhedonia is linked to problems with the complex and multifaceted psychological processes involved in reward processing. These include explicit processes of wanting, liking, and learning that are consciously perceived, and their implicit counterparts (denoted with quotation marks in the text) that are potentially unconscious, i.e., they can operate at a level not always accessible to conscious awareness. These components constantly interact and require careful scientific analysis to tease apart. Animal studies have provided measurements or behavioral procedures that are especially sensitive markers of each of the potentially unconscious processes (“wanting,” “liking,” and “learning”). Recently, some of these procedures have been successfully translated to human studies, thereby providing more objective behavioral measures to aid subjective self-report measures. In particular, recent developments of behavioral measures of “wanting” and “learning” are promising, while bias-free measures of “liking” reactions in humans have proven more difficult. **(B,C)** Examples of how a measure of “wanting” has been successfully translated from animal to human studies. **(B)** In animal studies, “wanting” can be measured by looking at how willing the animal is to exert effort in exchange for more palatable food rewards, for example by using a choice paradigm devised to look at effort-based decision-making (Salamone et al., 1994). **(C)** In human studies, “wanting” can be measured similarly, by looking at how much a participant is willing to work for a reward, for example by combining salient stimuli with key-press/force-grip procedures. The first study of this kind used key-presses to operationalize “wanting” as the effort participants exerted to increase or decrease viewing time of images of salient faces on a screen (Aharon et al., 2001). OFC, orbitofrontal cortex; ACC, anterior cingulate cortex; vmPFC, ventromedial prefrontal cortex; NAc, nucleus accumbens; PAG, periaqueductal gray; VP, ventral pallidum; VTA, ventral tegmental area; ACh, Acetylcholine; PIT, pavlovian instrumental transfer; EEfRT, effort expenditure for rewards task. Figure and figure legend modified and reprinted with permission from *Frontiers in Behavioral Neuroscience* (Rømer Thomsen et al., 2015).

The parsing of reward into wanting, liking and learning components was originally introduced by Robinson and Berridge (1993) in their influential incentive sensitization theory of drug addiction. The theory has received support in animal and human studies of drug addiction (Vezina and Leyton, 2009; Leyton and Vezina, 2013) and recently also in terms of behavioral addiction like Gambling Disorder (Leyton and Vezina, 2012; Rømer Thomsen et al., 2014). In Robinson and Berridge’s taxonomy they differentiate between core reactions that are not necessarily conscious (“wanting,” “liking,” and “learning”) and their conscious counterparts (wanting, liking, and learning, i.e., denoted without quotation marks; Berridge and Robinson, 2003; Berridge and Kringelbach, 2008). In other words, reward can be parsed into three main components—motivation, hedonic impact and learning—and each of these components consist of both conscious and unconscious subcomponents (see Figure 1A). For example, motivation consists of “(1) core incentive salience “wanting” processes that are not necessarily conscious (e.g., cue-triggered “wanting” for food or drugs) and (2) conscious desires for incentives or cognitive goals” (Berridge and Kringelbach, 2008, p. 2). Hedonic impact consists of “(1) core “liking” reactions that need not necessarily be conscious and (2) conscious experiences of pleasure, in the ordinary sense of the word, which may be elaborated out of core “liking” reactions by brain mechanisms of awareness” (Berridge and Kringelbach, 2008, p. 2). Similarly, learning (or learned predictions) include “(1) implicit knowledge as well as associative conditioning, such as basic pavlovian and instrumental associations and (2) explicit and cognitive predictions” (Berridge and Kringelbach, 2008, p. 2).

The subcomponents of reward constantly interact through the appetitive, consummatory and satiety phases of a pleasure cycle, but can be teased apart using systematic scientific analysis. Self-report measures can help identify the conscious components (wanting, liking, and learning) and provide valuable information on this level of processing. However, self-report measures are of course limited in their ability to capture unconscious processes, as well as in their ability to parse out contributions that may have been made by any of the unconscious processes, considering that these processes interact strongly together. In contrast, behavioral procedures from animal studies provide useful markers of the core “wanting,” “liking,” and “learning” reactions (Figure 1B). For example, “liking” reactions have been studied in rodents by measuring the affective orofacial expressions that are elicited in response to sweet tastes (Pfaffmann et al., 1977; Grill and Norgren, 1978a,b), and a number of procedures have been developed to study “wanting” in rodents, e.g., by measuring the effort exerted to obtain rewards (Salamone et al., 2007) or the ability of reward-related cues to act as motivational magnets (Wyvell and Berridge, 2000). In recent years, some of these animal models have been successfully translated to human studies and provide valuable behavioral measures of subcomponents of reward, which can complement traditional self-report measures (Figure 1C).

Overall, findings from animal and human studies applying these types of measures support the view that reward is a complex process consisting of several psychological components that correspond to partly dissociable neurobiological mechanisms (Berridge and Robinson, 2003; Berridge and Kringelbach, 2008, 2015). For example, there is strong evidence that dopamine plays an important role in “wanting,” but not in “liking” reactions. In animal and human studies where “wanting” and “liking” reactions have been systematically teased apart, specific manipulation of dopamine signaling has failed to shift “liking” reactions to rewards (Berridge and Valenstein, 1991; Peciña et al., 2003; Ward et al., 2012). In contrast, there is accumulating evidence that dopamine plays an important role in “wanting” processes. For example, elevation of dopamine has been shown to increase willingness to work for a food reward in rodents (Bardgett et al., 2009), while dopamine attenuation or blockade has the opposite effect (Cousins and Salamone, 1994; Salamone et al., 2007). Similarly, evidence from human studies suggests that amphetamine/L-Dopa-induced elevated dopamine increases subjective ratings of drug wanting, but not subjective ratings of drug liking during consumption (Leyton et al., 2002, 2007; Liggins et al., 2012). Recently, Wardle et al. (2011) provided evidence that elevated levels of dopamine increase willingness to work for reward in humans using a behavioral measure.

Building on the framework set forward by Berridge and Robinson (2003) suggesting that reward consists of multiple subcomponents of wanting, liking, and learning and recent proposals relating these processes to the appetitive, consummatory and satiety phases of a pleasure cycle (Kringelbach et al., 2012), we recently proposed to reconceptualize anhedonia as “impairments in the ability to pursue, experience and/or learn about pleasure” (Rømer Thomsen et al., 2015, p. 2). In this conceptualization of anhedonia, impairments in each of the subcomponents can lead to a malfunctioning pleasure system. Normally, wanting, liking, and learning processes are balanced over time, however this balance can be compromised by impairments in each of the components. Depending on which of the subcomponents are most affected, and how the components are affected, this can lead to distinct subtypes of anhedonia, that are associated with distinct imbalances of the pleasure system (Rømer Thomsen et al., 2015). For example, patients suffering from major depressive disorder often describe a diminished ability to pursue and experience pleasure, i.e., a progressive decrease in some (or all) of the reward components. In contrast, drug addiction can be characterized by excessive wanting for the drug of choice, which grows over time independently of drug liking. While anhedonia has traditionally been conceived as diminished responses (typically, diminished subjectively experienced pleasure), “our proposed framework acknowledges that both too much and too little activity in specific parts of the pleasure system can lead to pathological changes. This is for example illustrated in the excessive wanting for drugs in drug addiction or in disorders with hypersexuality” (Rømer Thomsen et al., 2015, p. 15).

It is important to note, that in this terminology (Rømer Thomsen et al., 2015) *pleasure* and *pleasure system* is not restricted to hedonic impact, but is instead used to encompass all of the phases of reward processing. This is in contrast to the dominating terminology, where *pleasure* is restricted to the hedonic impact of a reward, while *reward* is used to encompass all of the reward-related processes (see, e.g., Berridge and Kringelbach, 2008). In an attempt to avoid misunderstandings, I have changed the wording of our definition in the present paper to reflect the dominating terminology. Hence, anhedonia is defined here as “impairments in the ability to pursue, experience, and/or learn about *reward*.”

In line with our proposed reconceptualization of anhedonia, there has been a growing bulk of evidence suggesting that reward processing deficits are not restricted to impaired hedonic impact in psychiatric disorders typically associated with anhedonia. Findings from the past 5 years suggest that motivational and learning processes are impaired, e.g., in patients suffering from major depressive disorder (subsequently referred to as depression) and schizophrenia (Treadway and Zald, 2011; Fervaha et al., 2013b; Rømer Thomsen et al., 2015; Whitton et al., 2015). Part of this work is based on successful translations of animal models, thereby paving the way for validated behavioral paradigms that can supplement traditional self-report measures. These efforts are exciting and hold promise in terms of elucidating the role of subcomponents of anhedonia and the underlying neurobiological mechanisms across major psychiatric disorders. Here I review recent progress in the development and application of behavioral procedures that measure subcomponents of anhedonia in relevant patient groups (including patients suffering from depression, schizophrenia and addiction) and discuss implications for clinical assessment and treatment.

## Measuring Subcomponents of Anhedonia

In line with the generally accepted understanding of anhedonia as “decreased subjective experience of pleasure” [as per Ribot’s (1896) definition], the most popular way of measuring anhedonia has been self-report scales or questionnaires like The Fawcett–Clark Pleasure Scale (FCPS; Fawcett et al., 1983) or The Snaith–Hamilton Pleasure Scale (SHAPS; Snaith et al., 1995). The majority of these instruments are restricted to measuring subjective experiences of hedonic impact (i.e., liking), but some of the more recently developed questionnaires also include aspects of reward motivation (i.e., wanting). For example, The Temporal Experience of Pleasure Scale (TEPS; Gard et al., 2006) differentiates between anticipatory and consummatory experiences of pleasure, and The Sensitivity To Reinforcement of Addictive and other Primary Rewards (STRAP-R; Goldstein et al., 2010) measures liking and wanting of drug and non-drug rewards under various situations (e.g., current and hypothetical). Building on Robinson and Berridge’s incentive sensitization theory (Robinson and Berridge, 1993; Robinson et al., 2013), Lende (2005) developed a short Incentive Salience Scale that measures key aspects of drug wanting and has been used to predict addiction status.

While these instruments provide useful information about the conscious components of anhedonia, they are of course limited in their ability to capture unconscious components. Similar to research on reward (Berridge and Robinson, 2003; Berridge and Kringelbach, 2008), it is crucial to differentiate between conscious and unconscious components of anhedonia (Rømer Thomsen et al., 2015). Accumulating evidence suggests that we do not always know what motivates our behavior or brings us pleasure (Aharon et al., 2001; Winkielman et al., 2005; Moeller et al., 2009; Parsons et al., 2011), and there is convincing evidence that reward (also) affects our behavior on an unconscious level (Winkielman et al., 2005; Pessiglione et al., 2007, 2008; Aarts et al., 2008).

During the last 5 years a number of validated and useful behavioral procedures have been developed that can be used to measure impairments in the described subcomponents of reward (Figure 1). Of particular relevance are recent developments in behavioral procedures that can be used to measure impairments in the ability to pursue and learn about reward.

### Impaired Ability to Pursue Reward

A large number of animal models have been developed to study motivational processes by looking at behavior related to obtainment of rewards such as food. Of particular relevance here are models of the effort exerted to obtain rewards [e.g., by measuring how eagerly the animal runs for rewards in a runway (Berridge and Valenstein, 1991; Peciña et al., 2003) or its willingness to exert effort in exchange for more palatable food rewards (Salamone et al., 1994, 2007)] and of the ability of reward-related cues to act as motivational magnets [e.g., by measuring pavlovian instrumental transfer (Wyvell and Berridge, 2000, 2001)]. Recently, some of these models have been successfully translated to studies of humans and the reported findings offer intriguing information on the role of reward motivation across major psychiatric disorders.

The effort expenditure for rewards task (EEfRT), which was developed by Treadway et al. (2009), represent a good example of how a validated animal model of motivation (Salamone et al., 1994) can be successfully translated to human studies. The EEfRT is an effort-based decision-making task, where reduced reward motivation is operationalized as a decreased willingness to choose greater-effort/greater-reward over less-effort/less-reward options with varying probability (Treadway et al., 2009). Recently, the task has been applied to relevant clinical populations and provide evidence of reduced willingness to expend effort for rewards in patients with subsyndromal depression, first-episode depression and remitted depression, compared to controls (Treadway et al., 2012; Yang et al., 2014).

A recent longitudinal study of reward seeking behavior in individuals at risk of depression provides intriguing evidence of diminished reward motivation as a potential precursor of depression (Rawal et al., 2013). Adolescent offspring of depressed parents performed the Cambridge Gambling Task in order to measure betting behavior under different odds. Compared to healthy adolescents and adolescents with externalizing disorders, the adolescent offspring of depressed parents showed diminished reward seeking (i.e., betted less at favorable odds). Importantly, the magnitude of this diminished response predicted depressive symptoms, depression-onset and functional impairment 1 year later (Rawal et al., 2013).

Several recent studies have reported decreased willingness to work for rewards using the EEfRT or similar tasks in patients suffering from schizophrenia (Fervaha et al., 2013c; Gold et al., 2013; Barch et al., 2014). For example, Barch et al. (2014) reported that patients with schizophrenia were less willing to work harder when the size of the rewards increased or when the rewards were more probable, compared to control participants. Furthermore, among patients with schizophrenia, there was an association between choosing fewer greater-effort/greater-reward choices in the task and having more severe negative symptoms (self-reported) and worse community and work function (reported by caretaker; Barch et al., 2014).

Overall these findings are exciting and promising by providing strong evidence of reduced reward motivation across major psychiatric disorders. However, as we have previously stressed (Rømer Thomsen et al., 2015) participants are working for abstract rewards in these tasks, and not fundamental rewards (as in the animal models). Whether abstract and fundamental rewards are treated in the same way remains an open question, but emerging evidence suggests that there are important differences in the underlying brain processing (Sescousse et al., 2013a,b).

Other groups have used a related measure of reward motivation in humans by combining a key-pressing procedure with salient face stimuli (Aharon et al., 2001; Parsons et al., 2011). In these tasks, “wanting” is operationalized as the amount of work participants perform (i.e., by pressing a key) in order to change the duration they view images of adult/infant faces on a screen. Findings from these studies provide support for a dissociation of conscious liking ratings of salient face stimuli and the behavioral measure of “wanting” (Aharon et al., 2001; Parsons et al., 2011). For example, heterosexual males used more effort to keep female compared to male faces on a screen, but in a self-report task they rated the faces as equally attractive (Aharon et al., 2001). The use of salient face stimuli in combination with a key-pressing procedure represents a promising way to study possible impairments in the ability (or willingness) to work for fundamental social rewards in humans.

Moeller et al. (2009) have used a similar key-pressing paradigm in combination with salient drug-related stimuli and provide evidence for increased “wanting” of drug-related stimuli in drug addicted: cocaine addicted used more effort to view cocaine-related stimuli in a behavioral choice task, compared to control participants. Furthermore, they reported dissociation between a self-report measure of hedonic impact and a behavioral measure of motivation in cocaine addicted individuals: in the self-report task they rated pictures of pleasant scenes as more pleasant than cocaine-related pictures, however, in the behavioral choice task they did not show this preference (Moeller et al., 2009). These findings are in line with the incentive sensitization theory (Robinson and Berridge, 1993; Robinson et al., 2013) which argues that cue-triggered “wanting” of drug-related stimuli is enhanced in drug addicted individuals, and that these “wanting” processes are partly dissociable from “liking” processes in the brain, and in behavior. The reported dissociation between self-reported hedonic impact and a behavioral measure of motivation also reflects the impaired insight that characterizes drug addicted individuals (Goldstein et al., 2009; Moeller and Goldstein, 2014).

A related and promising measure of effort is the use of force-grip procedures which allows us to quantify various aspects of effort including; how much effort is exerted over time, how fast participants start to squeeze, or how fast the force is increased (Aarts et al., 2008). By combining force-grip procedures with subliminal priming paradigms it becomes possible to study motivational processes that we are not aware of (Pessiglione et al., 2007; Aarts et al., 2008). For example, it has been shown that subliminally priming of the concept of exertion (i.e., words such as “exert” or “vigorous”) can prepare people for forceful action, and when primes are accompanied with a rewarding stimulus (i.e., a consciously visible positive word) they are motivated to spend more effort (Aarts et al., 2008). In a similar set-up, Pessiglione et al. (2007) studied unconscious motivation with an Incentive Force Task, where the amount and reportability of monetary rewards participants could gain through physical effort varied. Pessiglione et al. (2007) reported that even when participants were unable to report how much money was at stake, they still used more effort for larger rewards. These paradigms have yet to be applied to samples of relevant patients, but they represent a promising way to investigate impairments in unconscious reward motivation.

Another important component of reward motivation is the ability of reward-related cues to capture our attention and act as motivational magnets. In human studies, one way of operationalizing the ability of certain stimuli to capture our attention is by using variants of the attentional blink paradigm. Studies using this type of measure provide evidence that drug addicted individuals display an attentional bias toward drug-related visual cues and that this bias is correlated with self-reported craving (Wiers and Stacy, 2006; Field et al., 2009; Tibboel et al., 2010). For example, heavy social drinkers have reduced attentional blink for alcohol-related stimuli, which is consistent with the hypothesis of enhanced attentional bias for salient drug-related cues (Tibboel et al., 2010). Recent evidence suggests that a similar mechanism is present in behavioral addiction like Gambling Disorder (Brevers et al., 2011a,b; Rømer Thomsen et al., 2014). For example, in an attentional blink paradigm problem gamblers exhibited enhanced processing of gambling-related cues compared to neutral cues (Brevers et al., 2011b), and in a change detection task problem gamblers were faster at detecting gambling-related stimulus changes compared to neutral (Brevers et al., 2011a). Taken together, these findings support the hypothesis of an attentional bias toward addiction-related stimuli in drug and behavioral addiction.

### Impaired Ability to Learn About Reward

There is an extensive literature from animal and human studies investigating the ability to learn from experiences with reward and punishment. Recently, some of these paradigms have been applied to relevant patient groups and provide evidence of impairments in the ability to learn about reward in patients suffering from depression and schizophrenia.

In a series of studies, Pizzagalli and colleagues have investigated impairments in the ability, or propensity, to develop a response bias toward stimuli that are more frequently rewarded than others using a probabilistic reward task (Pizzagalli et al., 2005, 2008; Pechtel et al., 2013; Vrieze et al., 2013). The task has been applied to patients with varying degree of depressive and anhedonia symptoms and findings from these studies consistently show evidence of impaired reward learning. In the first study, Pizzagalli et al. (2005) showed that in participants with low levels of depressive symptoms there was an increase in the response bias over time, which was not present in participants with high levels of depressive symptoms. Subsequent studies of clinical populations show evidence of diminished reward responsiveness in depressed patients compared with controls (Pizzagalli et al., 2008; Vrieze et al., 2013), in patients with remitted depression compared with controls (Pechtel et al., 2013), and in depressed patients with high vs. low levels of anhedonia symptoms (Vrieze et al., 2013).

Of relevance here are also studies using probabilistic learning tasks that differentiate between reward-guided and punishment-guided learning. So far, this type of paradigm has not been systematically applied to clinically depressed patients, but one study reported evidence of blunted reward- and punishment-guided learning in depressed patients compared with controls (Chase et al., 2010). More data is available from patients suffering from schizophrenia. Compared to controls, patients suffering from schizophrenia consistently show deficits in reward-guided learning, while findings regarding punishment-guided learning are conflicting (Waltz et al., 2007, 2011; Strauss et al., 2011; Gold et al., 2012; Yilmaz et al., 2012; Fervaha et al., 2013a).

Studies targeting impairments in unconscious reward learning are intriguing, considering recent evidence of reward learning occurring outside our awareness. Pessiglione et al. (2008) used a subliminal instrumental conditioning task, where cues predicting monetary reward or punishment are subliminally presented, and showed that participants develop a propensity to choose cues associated with reward, even though the cues are not consciously perceived. These findings support the notion that cues related to reward and punishment (also) affect behavior and decision-making processes on an unconscious level and underscores the need to study reward processing deficits on both conscious and unconscious levels. This type of paradigm has yet to be applied to relevant patients, but represents a promising method to study potential impairments in unconscious reinforcement learning.

In animal studies, the conditioned place preference (CPP) procedure has long been used to study the development of preferences for environments or stimuli which have previously been associated with rewarding drug intake through the process of classical conditioning (Tzschentke, 1998, 2007). Recently, Mayo et al. (2013) successfully translated the CPP procedure into a human drug conditioning task and showed that healthy participants develop a behavioral preference for cues that have been paired with drug intake (a dose of methamphetamine), compared with cues that have been paired with placebo. These findings were recently replicated and extended by including a broader range of measures of the conditioned drug response, including self-report, behavioral and psychophysiological measures. After the conditioning procedure, participants showed an increase in behavioral preference, positive emotional reactivity, and attentional bias toward the cue associated with drug intake, compared with the cue associated with placebo (Mayo and de Wit, 2015). This paradigm represents a promising method to study individual determinants of classical conditioning and is therefore highly relevant for the disorders discussed here. For example, this type of paradigm can shed light on individual risk factors in the development of sensitized responses to drugs/drug-related cues and blunted responses to other types of rewards (e.g., social, sexual, and sensory) in drug addiction, and similarly in behavioral addiction such as Gambling Disorder. This procedure is also highly promising in terms of studying deficient associative learning in patients suffering from depression and schizophrenia, preferably by using different types of rewards.

In line with the strong evidence suggesting that “wanting,” “liking,” and “learning” processes are dissociable in the brain and in behavior, it is important to note that the impaired reward learning reviewed here is not necessarily related to impairments in the ability to learn about “liking” (i.e., the hedonic impact of a reward), but could as easily be due to a reduced or modified sensitivity to the rewarding properties of the stimulus in the absence of “liking.” Future studies are needed to tease these differences apart.

### Impaired Ability to Experience Pleasure

While successful models have been developed to study aspects of reward motivation and reward learning in humans, it has proven more difficult to develop behavioral procedures that measure hedonic impact in humans. In animal studies, hedonic impact of pleasurable stimuli has been successfully studied by measuring affective orofacial expressions elicited by the hedonic impact of sweet tastes. Studies applying taste-reactivity paradigms have convincingly shown that sweet tastes elicit rhythmic licking of lips (i.e., facial “liking” reactions) and bitter tastes elicit gapes (i.e., facial “disliking” reactions) in rodents and human infants (Steiner, 1973, 1974; Pfaffmann et al., 1977; Grill and Norgren, 1978a,b; Steiner et al., 2001). However, these affective orofacial measures are not easily translated to (adult) human studies, because we learn to control and mimic orofacial reactions to food as we grow up.

The hedonic impact of other types of rewards (than food) appears to be easier to measure behaviorally, or physiologically. Although mostly taboo, there is an increasing interest in the mechanisms underlying sexual pleasures (Georgiadis and Kringelbach, 2012; Georgiadis et al., 2012), and a number of measures have been developed to quantify pleasure-elicited “liking” reactions to sexual pleasures, e.g., by measuring rectal pressure variability and self-reported level of sexual arousal (Georgiadis et al., 2006). Although impairments related to sexual activity and sexual pleasures are still taboo, they represent a promising area of research that can help shed light on impairments in hedonic impact in relevant patient groups, including patients suffering from depression, schizophrenia and addiction.

A number of studies have measured facial reactions to pictures of emotional facial expressions and there is some evidence of a blunted response to positive facial expressions in depressed patients (Bylsma et al., 2008). For example, Dimberg (1982, 1990) has used electromyographic (EMG) recordings to detect emotion-related facial movements and shown that we elicit distinct facial reactions in response to emotional facial expressions, which partly reflects a tendency to mimic the facial expression. Studies have shown that these reactions are elicited very rapidly (Dimberg and Thunberg, 1998) and even when participants are not aware that they are being exposed to facial stimuli (Dimberg et al., 2000). Although it is unlikely that all changes in facial musculature are related to emotion, EMG recordings of facial reactions may provide a way to investigate deficits in “liking” reactions to social pleasure (e.g., happy facial expressions). These rapid facial reactions have already been related to empathy (Dimberg et al., 2011), however more work is needed to confirm that they are in fact indicators of pleasure “liking.”

So far, the most popular way of measuring hedonic impact in humans has been to measure self-reported hedonic reactivity (i.e., subjective ratings of pleasure) to pleasant solutions and odors in a here-and-now setting. Surprisingly, the majority of studies report similar, or higher, pleasantness ratings in depressed patients compared to controls in response to sweet solutions (Amsterdam et al., 1987; Berlin et al., 1998; Scinska et al., 2004; Swiecicki et al., 2009; Dichter et al., 2010) and various odors (Steiner et al., 1993; Pause et al., 2001; Lombion-Pouthier et al., 2006; Scinska et al., 2008; Clepce et al., 2010). Similarly, evidence from studies of patients suffering from schizophrenia does not suggest that this patient group experiences lower levels of hedonic reactivity to pleasurable stimuli compared with controls (Heerey and Gold, 2007; Barch and Dowd, 2010; Strauss and Gold, 2012).

Interestingly, the same patient groups (depressed and schizophrenic) report diminished enjoyment in studies where they are asked to rate prospective, retrospective, or hypothetical experiences (McFarland and Klein, 2009; Watson and Naragon-Gainey, 2010; Strauss and Gold, 2012). One way of interpreting this discrepancy is that anhedonic patients retain core “liking” reactions, but do not cognitively value them in the same way as they did before (Dichter et al., 2010; Berridge and Kringelbach, 2011). This interpretation should however, be seen in the light of standard clinical examinations where depressed patients often present with behavioral characteristics that do not only imply impairments in cognitive evaluations of their experiences. For example, clinicians often report less smiling and less reactivity to stimuli (in general) which might reflect diminished “liking.” The disagreement—between laboratory based studies using taste-reactivity paradigms and clinical observations of patients—underscores the need to consider methodological aspects. The laboratory based studies reviewed here (where they failed to show diminished “liking” reactions to pleasurable solutions and odors in depressed and schizophrenic patients) were all based on self-reported ratings of hedonic impact. It remains an open question whether behavioral or physiological measures of “liking” will inform us differently.

## Implications for Assessment and Treatment

The generally accepted understanding of anhedonia as “diminished subjectively experienced pleasure” is reflected in current diagnostic classification systems. For example, in the DSM 5 anhedonia is one of two main symptoms needed for the diagnosis of depression and is defined as “decreased interest and pleasure in most activities most of the day” (American Psychiatric Association, 2013). In this definition of anhedonia, wanting and liking components are collapsed, which is in contrast to the accumulating evidence suggesting that these processes are in fact dissociable in the brain and in behavior. For example, findings from animal and human studies suggest that dopamine plays a crucial role in reward motivation (“wanting” and wanting), but not in hedonic impact (“liking” and liking; Berridge and Robinson, 2003; Berridge and Kringelbach, 2008).

Further, findings from affective neuroscience suggest that reward processing deficits are *not* restricted to impaired hedonic impact. As reviewed here, increasing evidence suggests that the ability to pursue and learn about reward is compromised in patients suffering from depression, schizophrenia, and drug/behavioral addiction (Treadway and Zald, 2011; Rømer Thomsen et al., 2015; Whitton et al., 2015). In contrast, it is less clear whether core “liking” reactions are in fact compromised in, e.g., depression and schizophrenia.

The growing evidence that reward processing deficits are not restricted to diminished experience of pleasure across major psychiatric disorders stresses the need to consider impairments in reward wanting and reward learning in clinical assessments. As a start, self-report instruments could be elaborated to reflect all phases of reward processing. The motivational aspect has already been included in some of the more recent questionnaires (e.g., the TEPS and the STRAP-R), but so far the learning component has been absent. Considering the growing evidence that unconscious components of reward affect our behavior, and are not always accompanied by conscious awareness (Berridge and Winkielman, 2003; Pessiglione et al., 2007, 2008; Aarts et al., 2008), it is highly debatable whether self-report instruments are sufficient in clinical assessments. Or whether they should be complemented by behavioral procedures. For example, behavioral measures of “wanting” could compliment self-report questionnaires in clinical assessments with advantage and help guide subsequent treatment. Depending on which subcomponents of reward processing are mainly affected, different medical treatments may be afforded. For example, depressed patients characterized by impaired ability to pursue pleasurable activities may benefit from medical interventions that target neurotransmitter systems such as the mesolimbic dopamine system and the opioid system, which have been shown to play a crucial role in reward motivation (Treadway and Zald, 2011; Soskin et al., 2013; Rømer Thomsen et al., 2015).

These insights are also relevant in terms of psychological treatment options. For example, cognitive behavioral therapy (CBT) has so far shown more promising treatment effects than pharmacological treatments in patients suffering from drug and behavioral addiction (Gooding and Tarrier, 2009; Potenza et al., 2011; Bullock and Potenza, 2012). In the context of addiction, CBT is expected to improve the individual’s control over motivation by increasing awareness of cues that trigger craving and by learning skills that enable new patterns of thinking and acting (Potenza et al., 2011). These efforts are important and efficiently target conscious feelings of craving. However, this type of cognitive intervention has limited efficacy in terms of targeting unconscious mechanisms. In particular, cue-induced craving reactions that occur outside our awareness are not likely to be targeted in CBT, but play an important role in maintaining the addictive behavior as outlined, e.g., by the incentive sensitization theory of addiction (Robinson et al., 2013). Hence, although CBT reduces some of the cognitive layers of responsiveness to drug cues, it is very likely that unconscious layers persist (Robinson et al., 2013).

Other types of psychological interventions may provide a way to target unconscious “wanting” (or “craving”) mechanisms, such as mindfulness based interventions that aim to improve the individual’s awareness of bodily and emotional signals (Garland et al., 2014). There is some evidence to suggest that mindfulness based interventions can reduce consumption and craving of a number of substances in substance users, although more randomized controlled trials are warranted (Chiesa and Serretti, 2014). For example, in a recent randomized controlled trial Tang et al. (2013) reported that brief meditation training reduced smoking by 60% in smokers who wanted to quit smoking, which was accompanied by increased activity in brain regions related to self-control and self-awareness. These findings foster hope that mindfulness based interventions can improve self-control and awareness of otherwise unconscious “wanting” reactions, and stresses the need to consider these types of treatments in combination with CBT, although more randomized controlled studies are warranted.

## Concluding Remarks

Ribot’s (1896) long standing definition of anhedonia as “the inability to experience pleasure” has been challenged following progress in affective neuroscience, and in particular following pioneering work suggesting that reward consists of multiple subcomponents that can be divided into the processes of wanting, liking and learning (Berridge and Kringelbach, 2008). Recent proposals to reconceptualize anhedonia as motivational or consummatory subtypes of anhedonia (Treadway and Zald, 2011), or as impaired ability to pursue, experience, and/or learn about pleasure (Rømer Thomsen et al., 2015) have paved the way for objective behavioral measures to complement traditional self-report measures of anhedonia. As reviewed here, a number of behavioral procedures have been developed that can be used to measure impairments in reward motivation and reward learning, while behavioral measures of hedonic impact have proven more difficult. Findings from studies applying these methods support the new conceptualizations of anhedonia by providing robust evidence that reward processing deficits are not restricted to impaired hedonic impact in major psychiatric disorders. Instead, there is increasing evidence of impairments in the ability to pursue and learn about reward in, e.g., depression and schizophrenia. This progress is essential for the purposes of identifying the underlying neurobiological mechanisms of anhedonia, and has important clinical implications for assessment and treatment of anhedonia. For example, self-report measures of anhedonia could be elaborated to reflect all phases of reward processing and it is debatable whether self-report measures of anhedonia are sufficient, or whether they should be complemented by behavioral measures in clinical assessments.

### Conflict of Interest Statement

The author declares that the research was conducted in the absence of any commercial or financial relationships that could be construed as a potential conflict of interest.
